# Learning Empathy Through Virtual Reality: Multiple Strategies for Training Empathy-Related Abilities Using Body Ownership Illusions in Embodied Virtual Reality

**DOI:** 10.3389/frobt.2018.00026

**Published:** 2018-03-22

**Authors:** Philippe Bertrand, Jérôme Guegan, Léonore Robieux, Cade Andrew McCall, Franck Zenasni

**Affiliations:** ^1^Frontiers VR Laboratory (CRI Labs), Institut Innovant de Formation par la Recherche, USPC, Centre de Recherches Interdisciplinaires, Paris, France; ^2^Laboratoire Adaptations Travail-Individu, Université Paris Descartes – Sorbonne Paris Cité, Institut de psychologie, Paris, France; ^3^BeAnotherLab Research, BeAnotherLab Association, Barcelona, Spain; ^4^Department of Psychology, University of York, York, United Kingdom

**Keywords:** embodied virtual reality, body ownership illusion, empathy-related, learning, training, prosocial behavior, bias, intergroup

## Abstract

Several disciplines have investigated the interconnected empathic abilities behind the proverb “to walk a mile in someone else’s shoes” to determine how the presence, and absence, of empathy-related phenomena affect prosocial behavior and intergroup relations. Empathy enables us to learn from others’ pain and to know when to offer support. Similarly, virtual reality (VR) appears to allow individuals to step into someone else’s shoes, through a perceptual illusion called embodiment, or the body ownership illusion. Considering these perspectives, we propose a theoretical analysis of different mechanisms of empathic practices in order to define a possible framework for the design of empathic training in VR. This is not intended to be an extensive review of all types of practices, but an exploration of empathy and empathy-related phenomena. Empathy-related training practices are analyzed and categorized. We also identify different variables used by pioneer studies in VR to promote empathy-related responses. Finally, we propose strategies for using embodied VR technology to train specific empathy-related abilities.

## Introduction

This work combines studies and reviews from research in cognitive science, psychology, education, medicine, the arts, and virtual reality (VR) to address one specific topic: the potential use of VR for learning empathy-related abilities. The article is divided into three sections which address the following questions: (A) What empathy-related abilities should be enhanced? (B) What are good training strategies to enhance these abilities? (C) What is the best use of VR to enhance these abilities?

In section (A), we will thus explore empathy-related phenomena that can be trained to facilitate healthy and prosocial responses. Therefore, we will highlight strategic abilities to be enhanced (intergroup empathy, compassion, perspective taking, self-regulation) and to be avoided (personal distress). In section (B), we will focus on methods for training empathy without the necessary use of technology. Finally, in section (C), we will explore the potential of VR in promoting empathy, presenting advances in the use of immersive embodied virtual reality (EVR) that has shown efficiency in enhancing empathy-related capacities and their potential in enhancing empathy-related training strategies. We will also present one example of an artistic work that makes use of a wide combination of these techniques to address empathy-related experiences outside the context of a lab. To conclude, we will propose a framework that integrates the critical points identified in the three sections above in the design of new learning applications using embodied VR for promoting empathy-related abilities.

## Section (A): Empathy and Empathy-Related Abilities

### Definition and Description of Empathy

In recent years, a diverse range of disciplines have investigated the roles played by the presence and absence of empathy and empathy-related phenomena in affecting prosocial behavior and intergroup relations. Empathy can be defined as feeling the same emotion as another observed individual without mixing it with one’s own direct experience (de Vignemont and Singer, 2006; Decety and Meyer, 2008; Singer and Lamm, 2009; Decety, 2010). Empathy is deeply related to social bonding and allows one to feel compelled to help another (Decety, 2010). This affective state is produced by the interaction of multiple neural circuits related to motor, cognitive, emotional, motivational and behavioral functions (McCall and Singer, 2013). These different functions are referred to in this article with the broad term empathy-related phenomena. They include perspective taking, affective empathy, empathic distress, empathic concern and altruism, among others. Empathy-related phenomena are crucial for successful social interactions, allowing one to better understand the other, learn from other’s actions, and eventually provide help. Therefore, they may help societies to evolve through collaboration (Decety, 2010; McCall and Singer, 2013).

On the one hand, a healthy collective empathic process can help individuals and societies to hold behaviors and cultural beliefs consistent from the moral perspective of maintaining human rights (Decety, 2010). On the other hand, some unhealthy empathic responses may lead individuals to personal distress and burnout (Hojat et al., 2009; Klimecki et al., 2013) and antisocial behavior such as avoidance (Batson et al., 1987; Eisenberg and Fabes, 1990) and unfairness toward outgroup members (Decety, 2010).

Empathic responses emerge at a very young age. Altruistic responses to victims of stress, for example, can be found in babies as young as 12 months (Warneken and Tomasello, 2009). The level of empathy and prosociality increases from 14 to 36 months, with prosociality mainly affected by environmental effects (Knafo et al., 2008). By that time, children develop a better sense of self and other awareness, and throughout childhood and adolescence they develop the emotion regulation abilities (Decety, 2010). As children grow, they develop a more complex use of relational and contextual factors, goals, and beliefs (Harris, 1994), affecting their capability for mature empathic processes (Decety, 2010).

Empathic abilities are profoundly related to familiarity and affiliation to a group. We tend to develop empathy more easily toward the ones who are familiar to us or that we identify as part of our group (Avenanti et al., 2010). In fact, the lack of intergroup empathy is a phenomena deeply embedded in the way our society perceives and interacts with outgroups (Kubota et al., 2012; Eres and Molenberghs, 2013; Amodio, 2014). This lack of empathy is related to negative bias and stereotypes at implicit levels as well as to more explicit forms of racism and aggression (Cosmides et al., 2003).

### Defining Empathic Phenomena Through Their Processes and Expressions

“*Try to walk a mile in another person*’*s shoes*.” This proverb, found in many cultures in the world, suggests a way to help us understand each other better, and relates to several empathy-related responses. Imagine if we try to follow this proverb literally and walk a mile in the shoes of someone in need. First, we would (a) *move our own body, copying* the other person’s movements. Then we would (b) *feel distressed* for walking in their place and facing their needs. Doing so, we would (c) *understand* what the other person is going through and would (d) *understand* what they are *feeling*. Also, we would (e) *feel* the emotions that the other feels in their trajectory. After doing so, we may feel the (f) *desire to help this person*. This desire, could (g) *drive us to actually* help the other, even if that action is costly to our self.

In the same order, under a psychological or neuroscientific perspective we can identify the following empathy-related phenomena in this proverb. (a) *Mimicry* is a tendency to synchronize the affective expressions, vocalizations, postures, and movements of another person (Chartrand and Bargh, 1999). (b) *Empathic Distress* is when one is personally distressed by the distress of another person (Batson et al., 1987). (c) *Perspective taking* is the cognitive ability of imagining the perspective of others (Reniers et al., 2011; Myszkowski et al., 2017). (d) *Online simulation* is the ability to predict other people’s emotions (Reniers et al., 2011). Perspective taking and online simulation are sub-factors of cognitive empathy (Reniers et al., 2011). (e) *Affective empathy* (or simply “empathy”) means experiencing an isomorphic feeling in relation to others with a clear differentiation between self/other, knowing that the origin of the emotion comes from the other (de Vignemont and Singer, 2006; Singer and Lamm, 2009). Therefore, affective empathy is related to the emotional engagement of the observer with the situation of the emoter. (f) *Compassion* is an emotional and motivational state of care for the wellbeing of the other (McCall and Singer, 2013). Finally, (g) *altruism* is characterized by the prosocial behavior of helping others at a cost to the self (de Waal, 2008).

Affective empathy is one specific emotional response that tends to interact with motor, cognitive, and behavioral phenomena (McCall and Singer, 2013). Enhancing abilities in one domain can spillover to benefits in others (McCall and Singer, 2013). For example, empathy increases mimicry (Chartrand and Bargh, 1999). Affective empathy can also be enhanced by cognitive empathy (Batson et al., 2014), which allows individuals to have an accurate understanding of the situation and the feelings of the other (referred to as empathy accuracy; Main et al., 2017). Since empathy creates an isomorphic response to another person’s feelings, an empathic response to the distress of others can cause overwhelming distress in the observer. This is defined as empathic distress (Eisenberg and Fabes, 1990) and can lead to “an egoistic motivation to reduce stress by withdrawing from the stressor” (Decety, 2010) and therefore lead to social avoidance. In medical patient relations, for example, empathic distress is related to burnout (Hojat et al., 2009; Klimecki and Singer, 2012; Zenasni et al., 2012). On the other hand, moderate levels of distress may be necessary to drive one to feel empathic concern (Decety, 2010), which is the desire for the wellbeing of others and, therefore, the desire to help. Empathic concern is also described as compassion and relates to a loving-kindness emotion that is not isomorphic in relation to the emoter (Singer and Steinbeis, 2009; Klimecki et al., 2013). This means that the observer does not feel the same as the emoter but is still aware of self-other differentiation (McCall and Singer, 2013). Empathic concern may drive one to altruistic behavior (i.e., to help another person in need even at personal cost) (Batson, 2011; McCall and Singer, 2013).

As we will discuss in the next section, all of these empathic processes are affected by specific moderators relating to awareness of others, awareness of oneself, group identity, motivation, and behavioral affordances.

### Moderators of Empathic Responses

Several factors moderate and even completely block empathic responses. These include psychological processes that preempt empathy, for example perceptions of unfairness (Decety and Cowell, 2015) and dehumanization—associating others as machines, non-human animals or as individuals with no human rights (Bain et al., 2013). In this section, we will discuss different moderators of empathic responses.

#### Familiarity, Affiliation, and Similarity Trigger Enhanced Empathy Toward Ingroups and Reduced Empathy Toward Outgroups

We tend to feel greater empathy toward familiar individuals or individuals whom we perceive to be similar to us. This response is part of a general favorability toward similar others (Decety, 2010). This effect causes what can be called enhanced empathy toward ingroup members, due to positive biases toward them (Mathur et al., 2010). Conversely, it can lead individuals to behave unfairly toward outgroup members by comparison (Decety and Cowell, 2015).

The separation between “us” and “them” is not static and can be very subtle. On the one hand, group affiliation and ingroup empathy can be predicted by race (Chiao and Mathur, 2010). Phenotypes—gender, age, skin color—are clearly known to create intergroup barriers. On the other hand, environmental or social factors such as a mixed race group may predict social biases (Van Bavel and Cunningham, 2009) and empathic responses (Weisbuch and Ambady, 2008). For example, individuals from different races may present ingroup responses for others from different races but from the same basketball team (Weisbuch and Ambady, 2008). A classic behavioral experiment showed that football fans primed with the idea of interteam competition were less likely to help an individual in need if he wore a t-shirt of a rival team. Conversely, when primed by the idea of a uniting passion for football, they were more likely to engage in altruistic behavior, even for rival supporters (Levine et al., 2005). Another bonding factor (also used as a strategy to overcome biases) is to consider the observed individual separated from the group, humanizing them. In that way, the observer may present an empathic response to one observed individual, but not to his/her group (Kubota et al., 2012).

#### Negative Intergroup Evaluation Block Empathic Response Targeted at Outgroups

Explicit or implicit negative evaluation of an emoter considered an outgroup member can decrease or completely block any empathic response in the observer what can be caused by different constructs, such as negative stereotypes, negative biases and anxiety. These mechanisms can have a very high-speed automatic process that demands low cognitive resources whether the individual is aware or not (Amodio, 2009). Subjects with implicit, but not explicit, bias against outgroup members present reduced sensorimotor resonance to vicarious pain targeted to outgroup members (Avenanti et al., 2010). Stereotypes, biases and anxiety may block one’s empathic responses even among individuals with egalitarian values (Correll et al., 2002; Decety, 2010). Categorization of social groups, activation of group stereotypes and use of those stereotypes to form impressions of others is a common practice adopted by social perceivers that can vary in terms of chronic and situational, as well as cognitive and motivational factors, augmenting or reducing stereotypes to form a judgment (Quinn et al., 2003). This categorical thinking based on coded predictions saves cognitive resources on real life interactions, that are usually complex (Macrae and Bodenhausen, 2000). That means that our predictions are shaped by simplified associations present in our long-term memory based on stereotypes, fears, and personal experience (Macrae and Bodenhausen, 2000). Regarding empathy, coded predictions are deeply related to stereotypes (semantic associations) and to biases (visceral categorizations), that can be either negative (when related to outgroups) or positive (when related to ingroup members), respectively, blocking or enhancing one’s empathic response toward another (Amodio, 2009). Intergroup interactions are also modulated by the relation of power (resources) and prestige between groups (Fiske et al., 2016) and the perception of competition between groups may also intensify intergroup biases (Esses et al., 2001).

### Perceived Unfairness Blocks Empathic Responses in Men

Perceiving another individual as unfair appears to moderate one’s empathic response to them. Neuroimaging studies have shown that empathy-related responses were significantly reduced in males when observing an individual who had behaved unfairly receiving pain (Singer et al., 2006). Instead of feeling the other’s pain, this activated an area of the brain related to reward, revealing the pleasure in punishment of unfair others. Curiously, this effect was not observed in women. Even when women disliked one person due to a perceived unfairness they still presented vicarious pain responses.

#### Self-Awareness Allows One to Identify Feelings and Their Origins, Self-Affiliation, and Social Values

We refer to self-awareness as a combination of different phenomena related to the perception of one’s own emotions, body, and semantic constructs of themselves. First, the awareness of the external origin of one’s own vicarious emotions is what differentiates empathy from emotion contagion (de Vignemont and Singer, 2006). Furthermore, individuals vary in their ability to perceive and identify their own emotions and sensory states. For example, alexithymia, has been found to reduce empathic responses (Bird et al., 2010; Bernhardt and Singer, 2012). Second, the plasticity of the perception of the bodily and conceptual self may interact with other modulators such as affiliation, similarity and familiarity enhancing empathy by letting observers to feel more identified with emoters (for a complete review on plasticity of self-perception see Farmer and Maister, 2017).

#### Emotion-Regulation: Enables Empathic Concern Instead of Empathic Distress

Emotion regulation can act as a key factor to allow individuals to self-regulate their own stress, in order to direct affective empathy into empathic concern instead of personal distress (Decety, 2010; McCall and Singer, 2013). Emotion-regulation is a complex top-down process that allows individuals to initiate, inhibit or modulate their own emotional state or behavior in response to a given situation. Dispositional differences in the abilities of individuals to regulate their emotions have been shown to relate to differences in the tendency of experiencing empathic concern or distress (Rothbart et al., 1994). For example, children with a greater ability to focus or shift their attention have been found more able to present compassion responses; by modulating their negative vicarious emotions, children can keep their emotional arousal at a moderate level, thereby controlling their distress (Eisenberg and Eggum, 2009).

#### Self-Regulation of Behavioral Expressions: Control Inconsistencies (Errors) Between Goals and Biases (as Well as in Conditions Mentioned in Item Negative Intergroup Evaluation Block Empathic Response Targeted at Outgroups)

Implicit biases are likely to emerge even among individuals with egalitarian goals (Devine, 1989; Monteith, 1993; Greenwald et al., 1998; Devine et al., 2002; Amodio et al., 2003; Cunningham et al., 2004). Analysis of the self-regulation of stereotyping suggests that, in these cases, individuals experience guilt and redirect their behavior (Monteith, 1993; Czopp et al., 2006). Furthermore, the motivation to be consistent with egalitarian goals (Bargh et al., 2001; Moskowitz and Ignarri, 2009) can help individuals control non-empathic behaviors. Nevertheless, a great deal of research has shown that successfully suppressing unwanted thoughts or emotions is exceedingly difficult (Wegner and Erber, 1992; Gross and Levenson, 1993; Neil Macrae et al., 1994; Wegner, 1994; Monteith et al., 1998). The same likely holds for the self-regulation of behavior (e.g., Monteith, 1993; Monteith et al., 2002; Monteith and Mark, 2005). These self-regulatory mechanisms are based on two different components: monitoring and operating (e.g., Wegner, 1994). While monitoring operates in a relatively automatic manner and does not require deliberative thinking (Amodio et al., 2004), operating processes to control behavior require high motivation, attention and sufficient cognitive resources, and may not occur in complex situations, distraction, or cognitive load (Gilbert and Hixon, 1991; Spencer et al., 1998). Such situations may lead to a reduction in controlled empathic responses.

#### Interpersonal Skills Allow One to Develop Empathic Accuracy

Empathic or non-empathic processes can also be defined by the quality of the interaction between emoter and observer (Main et al., 2017). On the one hand, to be properly recognized by the observer, the stimuli must be clear. That means that emoters with good communication skills and expressivity are more likely to trigger empathic responses (Greenson, 1960; Ickes et al., 1997; Halpern, 2001; Hollan, 2008, 2012). On the other hand, from the perspective of the observer, present attention and openness to understand the empathic stimuli are also needed (Main et al., 2017). Real time tuning to the mental states of others is needed to generate an accurate empathy response.

#### Motivation, Power, and Skills Modulate Altruistic Behavior

Even when feeling empathy, individuals may not express empathic behavior such as altruism. Altruistic motives (other-oriented rather than self-oriented motivation) are related to the amount of help one individual may offer to someone in need. Helping behavior is also related to the skills and abilities of individuals facing helping-tasks, as well as the power to offer effectiveness help (Clary and Orenstein, 1991). For a review on different theories of altruism see Feigin et al., 2014.

Table 1 summarizes highlighted empathic processes, abilities, and modulators discussed in section (A).

**Table 1:** Highlighted empathic abilities to be developed: empathy-related dimensions that may be promoted to develop prosocial empathic expressions.

Optimal empathy-related process	Highlighted empathy-related abilities	Moderators of empathic processes and expressions
Positive intergroup interaction and evaluation	Openness for intergroup empathy	Familiarity, affiliation, similarity within outgroup members
		Bias, threat evaluation, stereotype and categorical thinking related to outgroup members; perceived fairness

Awareness of the self	Emotional self-awareness, plasticity of self-perception	Self-other distinction; emotion recognition; plasticity of bodily and conceptual self-perception

Awareness of the other	Affective empathy	Emotional engagement
	Cognitive empathy	Perspective taking, online simulation
	Empathy accuracy	Dialog skills; present attention
	Empathic distress moderation	Emotion regulation

Positive emotional motivation	Compassion (or empathic concern)	Loving-kindness toward the other

Altruistic behavior	Altruism	Motivation, power and skills for helping
		Self-regulation of behavioral expressions

## Section (B): Existent Strategies for Learning Empathy-Related Abilities

### Definition and Description of Potential and Actual Abilities

When discussing for the training of empathic abilities it is important to make a clear distinction between potential and actual skills. In other words, one individual may have the potential to be empathic but not necessarily have the optimal environmental conditions for expressing empathy. This is clearly described by some models such as the Gagné’s Differentiated Model of Giftedness and Talent (Gagné, 2013). The main asset of the model is to remember that any potential (the natural ability), whatever its original level, does not necessarily develop spontaneously. It must be underpinned by an appropriate environment, dispositional factors and support. The environmental and intrapersonal factors are called catalysts. In the case of empathy, educational methods should stimulate learners through specific catalysts (e.g., emotionally safe environment, multicultural, collaborative, dynamic, engaging activities to stimulate openness, facilitators to support the learning process) and through the development of specific skills (e.g., perspective taking training, compassion practices, self-regulatory methods, reflexive thinking, social and emotional skills). In this section, we will discuss three methodologies that include catalyst factors within educational contexts and three methodologies that directly train specific empathic abilities.

### Methodologies for Empathy-Related Learning in Educational Contexts

#### Social and Emotional Learning (SEL): A Long-Term Holistic Approach

Research shows that many students lack social–emotional skills and become disengaged as they progress through school, which interferes with academic performance, behavior, and health (Klem and Connell, 2004). In one study from 2006 with 148,189 American students from sixth to twelfth grade, between only 29 and 45% reported having social competencies such as empathy, decision making, and conflict resolution skills; 71% indicated that their school did not provide an encouraging environment (Durlak et al., 2011). To address these topics, the process of SEL was created to enable learners to identify and manage their emotions, motivations, decisions, and social relations (Elias et al., 1997) through self-awareness, self-management, social awareness, relationship skills, and responsible decision making (Collaborative for Academic, Social and Emotional Learning, 2005—casel.org). These skills relate to some of the empathic phenomena discussed in section (A), specifically regarding perspective taking, empathic accuracy, and emotion regulation. A systematic and well implemented SEL program can allow students to learn, model, and practice these skills and apply them to diverse situations such that they become a part of their repertoire of behaviors (Ladd and Mize, 1983; Weissberg et al., 1989). A meta-analysis of SEL practices (Durlak et al., 2011; Taylor et al., 2017) showed that the conceptual model of targeting various social and emotional assets can be associated with significant improvement in students’ social and emotional skills, as well as academic performance and less risky behaviors. These empirical findings are in line with the educational literature on how intrapersonal and interpersonal competencies—such as self-regulation, problem solving, and relationship skills—may enhance academic performance and prosocial behavior of students. To have an optimal impact and to offer conditions for students to improve learning, behavior and wellbeing, SEL approaches use two great catalysts: long-term training, and a safe environment in a “whole school approach.” The latter engages all members of the school community (management, staff, students, parents, broader community) to work together and to create a safe environment through a sense of belonging and cohesion (Durlak et al., 2011).

#### Constructivism: Instrumentalization of Reflexive Thinking

Constructivism combines a series of approaches and methodologies developed to empower students by helping them to learn more than just content, but also to learn how to learn, and to develop cognitively, socially and emotionally (Karagiorgi and Symeou, 2005). It has been shown that constructivist learning environments enhance student’s emotional and social abilities, such as self-regulation and perspective taking (Karagiorgi and Symeou, 2005). Some of its strategies are specifically interesting for training perspective taking by offering realistic and plausible stimuli for understanding another’s point of view. These representations of reality avoid oversimplification by representing the natural complexity of the world. They present authentic tasks that contextualize rather than providing abstract instruction. They furthermore provide real world, case-based learning environments, rather than predetermined instructional sequences. Altogether, they enable context, and content, dependent knowledge construction (Jonassen, 1994). Constructivism approaches regularly involve cooperative tasks. Educators play the role of facilitators engaged in dialog with students, rather than monologs, which is especially interesting for instrumentalization of reflexivity, letting learners find their own answers, and leading with their misconceptions (Karagiorgi and Symeou, 2005).

#### Safe Environment for Positive Intergroup Interactions: Facilitating Positive Connections

To explore the interaction between observer and targeted outgroups can be an effective way to overcome fear and stereotypes. Intergroup contact (Allport, 1955) can improve positive intergroup attitudes when focused on equal status, cooperation, and common goals (Tropp and Page-Gould, 2015). Affective connection to outgroups (liking one outgroup individual) can decrease prejudice by stimulating perceptions of familiarity (Zajonc, 1968, 1980). Friendship with outgroup individuals may also reduce prejudice through prosocial contact (Allport, 1955; Collins and Ashmore, 1970). For example, one quasiexperimental study combined content and intergroup interaction (Rudman et al., 2001) in a 14-week conflict seminar held by an African American male professor. The intervention reduced prejudice and stereotyping among participants, a pattern that was mediated by cognitive factors (the content of the seminar) but also by affective experiences (liking the professor). Similarly, by combining intergroup interaction with perspective taking training (Malhotra and Liyanage, 2005), a 4-day intergroup workshop conducted between Sri Lankan Singhalese and Tamils resulted in enhanced empathy toward outgroup members 1 year after the intervention. Although intergroup interaction may be effective when it leads to appraisal, this approach presents some strong limitations. In real life, individuals tend to interact within their own social group, missing the opportunity to accumulate personal experiences *via* social interactions with outgroup members. Moreover, prejudiced people avoid intergroup contact (Pettigrew, 1998). In fact, obligatory courses in diversity have been reported to enhance racial bias in comparison to control students (Bigler, 1999). After enforced multicultural training, individuals with high external motivation but low internal motivation actually responded in anger to behavioral measures targeting outgroups (Plant and Devine, 1998). Furthermore, adult interventions based on the contact hypothesis (Allport, 1955) rarely diminish bias against outgroups in general, but only improve responses to outgroup members present in these interventions (Hewstone, 1996). Moreover, interventions based in “color-blind” strategies (encouraging individuals to suppress their category-based stereotypes in favor to more personalized judgments) have been shown ineffective (Wolsko et al., 2000), and actually enhance negative bias showing a backfire effect (Schofield, 1986; Wegner, 1994). Together these data suggest that although intergroup interactions may reduce bias, interventions along these lines must account for the motivations of the individual.

Table 2 summarizes the methodologies for educational contexts reviewed in this section, highlighting respective strategies on promoting catalysts factors and natural abilities.

**Table 2:** Highlighted educational approaches for empathy-related learning through environmental stimuli and enhancement of specific empathy-related abilities.

Examples of methodologies in educational contexts	Highlighted catalyst empathic stimuli	Highlighted natural empathy-related abilities
Social and Emotional Learning (SEL)	Long-term training; sense of belonging and cohesion within school, families, and communities	Instrumentalization of self-regulation, social, and emotional skills

Constructivism	Cooperative dynamics; real world case based and contextual knowledge; educators as facilitators	Instrumentalization of reflexive thinking

Safe environment for positive intergroup interaction	Engaging voluntary activities; cooperation, equal status among participants; supports familiarity and friendship among participants	Training on perspective taking and conflict management.

### Training Methods for Empathic Abilities

In this section, we provide examples of three different mind training methods to enhance abilities related to empathy. These approaches were selected because they offer insights for the development of new training methods using VR.

#### Role Playing: Enhancing Cognitive Empathy and Emotional Development

Playing the role of a movie character, such as Superman, can be one of the most effective types of play for developing perspective taking (Whitebread et al., 2012). As previously discussed, perspective taking is fundamental for understanding accurately the point of view of another person. Accordingly, perspective taking of an outgroup individual decreases explicit and implicit stereotypes toward the individual and increases positive evaluations toward their group (Galinsky and Mussweiler, 2001). Kwon and Yawkey (2000) also show interesting relations between emotional development and role-playing. Using basic foundations of psychoanalytical and learning theories they discuss how emotional development can be enhanced through role-playing tasks that enhance skills such as interactive levels of expression, control and modeling of emotion, and emotional intelligence. Role playing has also been used in digital games focused on empathy. *Real Lives* allows players to inhabit the lives of individuals around the world. In a quasiexperimental study with high school students in three schools in the USA (Bachen et al., 2012), students who played *Real Lives* as part of their curriculum, expressed more global empathy (observed in their identification with the characters played) and greater interest in learning about other countries.

Besides education, role playing techniques have been used in therapeutic contexts, conflict mediation, restorative justice, and many other fields. In each of these fields, different practices are proposed to help participants visualize events and conflicts from the perspective of others. These practices use physical dynamics (e.g., changing seats with another participant) and narratives related to real life (e.g., reporting on conflict from the protagonist’s point of view) to provide multisensory experiences. Successful perspective taking tasks tend to involve more immersive techniques, such as writing an essay about the other’s perspective (Todd and Burgmer, 2013) or taking the role of an outgroup member in a computer game (Gutierrez et al., 2014).

#### Mindfulness Training: Enhancing Several Empathic Processes

In recent years, mindfulness practices have been getting more attention due to putative therapeutic benefits for depression, anxiety, and chronic pain (e.g., Baer, 2003; Grossman et al., 2004; Galante et al., 2014) and even burnout in the workplace (Krasner et al., 2009). The term mindfulness has been used to describe states, traits, psychological functions, cognitive processes, and different types of meditation practices or intervention programs (Vago and Silbersweig, 2012). In this article, we use mindfulness to describe secular methods of mental training such as Mindfulness-Based Stress Reduction (Kabat-Zinn, 1982), Mindfulness-Based Cognitive Therapy (Segal et al., 2002), and Compassion Cultivation Training (Jazaieri et al., 2013). Each of these methods are driven by different goals, and the article will address specific outcomes relating to empathy-related phenomena, in enhancing natural abilities such as perspective taking and compassion (Klimecki et al., 2013; Hildebrandt et al., 2017) and to address modulators of empathy such as anxiety control and non-judgmental thinking (Lueke and Gibson, 2014). Mindfulness practices often help the subject to focus their attention to their own breath (Bishop et al., 2004), suggesting that interoceptive awareness may be used as an effective strategy in developing empathy-related abilities. Another common approach in many mindfulness practices is to observe thoughts without suppressing them (Bishop et al., 2004). Mindfulness practices enable individuals to focus their attention on automatic cognitions, such as implicit race biases, and can therefore modulate explicit social judgments and behaviors (Payne, 2005). Many mindfulness approaches consist in daily practices that are intended to generate positive outcomes after months of practice (Hildebrandt et al., 2017), but some studies have shown significant benefits after one single intervention (Klimecki et al., 2013; Lueke and Gibson, 2014). Using implicit association tests (Greenwald et al., 1998), Lueke and Gibson (2014) showed that one single 10 minute intervention of mindfulness—aiming to let subjects simply observe thoughts and events in a nonjudgmental way—helped individuals reduce biases against outgroups. Mindfulness practices to develop kindness and compassion have also been associated with lower levels of implicit bias (Kang et al., 2014) and to enhancing altruistic motivations (Condon et al., 2013). Different mindfulness practices appear to have different effects in these domains. Hildebrandt and colleagues (Hildebrandt et al., 2017) analyzed self-reported effects of mindfulness training comparing different types of interventions, focusing, respectively, on present attention, perspective taking, and compassion motivated practices. Results showed that the present attention training was able to significantly increase ratings in self-reported mindfulness, but not improve reports of perspective taking and compassion. Conversely, interventions focusing on perspective taking and compassion enhanced subjects self-reported ratings in all three domains. Specifically, the compassion motivated practice revealed the broadest effects, leading to enhanced abilities of present attention, perspective taking, compassion, and self-compassion, showing the great potential of cultivating compassion mind states to enhance empathy-related abilities. Another study by Klimecki et al. (2013) tested the behavioral and neural effects of one single 6-h training session on compassion-based therapy. The results showed an increase in positive affect, even in response to others’ suffering. The phenomenon was observed in brain areas related to positive evaluation (Kringelbach and Berridge, 2009), love (Bartels and Zeki, 2000, 2004; Aron et al., 2005) and affiliation (Vrticka et al., 2008; Strathearn et al., 2009). Taken together, these studies reveal how mindfulness practices can have powerful effects in training empathy-related abilities.

#### Implementation of Egalitarian Goals: Enhancing Self-Regulation of Behavioral Expressions

Even when biases and stereotypes initially preempt one’s empathic responses, it is possible to upregulate empathy to adopt behaviors in line with internal and social values. At least three different strategies can be implemented in these cases (for review on the strategies listed see Kubota et al., 2012):

- Mental-scanning of immediate responses: self-awareness of non-empathic responses (biases, stereotypes, anxiety) does not require high cognitive resources and can be learned and practiced. Recognizing these responses is the first step in controlling non-empathic expressions. When non-empathic responses are not identified, it is impossible to control them.
- Internal goals: defining egalitarian personal goals allows one to suppress non-empathic expressions and enhance empathic expressions. This strategy can be practiced by individuals with egalitarian goals but is efficient only when high cognitive resources are available.
- Social goals: social goals may function like internal goals, also requiring high cognitive resources, but with less power to control an individual’s expressions. Social goals such as egalitarian goals or social moral values against racism or prejudice tend to be somewhat more effective in individuals concerned about their social image and when being observed by others.

Different studies have explored priming methods to implement internal and social goals in order to overcome negative responses to outgroups, changing the attitudes and perceptions of outgroup members. Three interesting approaches are listed below:

- Exposure to non-stereotypical associations (Kawakami et al., 2005).
- Tasks demanding focus on the individual (individuation), rather than the social group or in the inhibition of stereotyping (Mason and Macrae, 2004; Quinn et al., 2009; Hutter et al., 2013).
- Exhaustive practice of negation (saying “no” to) stereotypes (Kawakami et al., 2000).

Although these methods were found to be efficient in lab conditions, they may not be feasible or effect in everyday life. For example, they may not be effective when the observer is repeatedly exposed to stereotypical and biased information in their everyday environments.

Table 3 summarizes training methods discussed, highlighting the environmental context in which they can be applied, and the natural abilities involved.

**Table 3:** Highlighted training methods for natural empathic abilities and respective environmental contexts where these trainings can be applied.

Highlighted training methods	Environmental contexts	Highlighted natural empathy-related abilities
Role playing: perspective taking practices	Developed by humans in early child hood as one type of play, can be applied in several contexts	Cognitive empathy, emotional development

Mindfulness training: practices of present attention, perspective taking, and compassion	Developed in training sessions, can be applied in real life as a long self-managed method	Present attention; non-judgmental thinking, interoceptive awareness, perspective taking, compassion, control of anxiety

Implementation of egalitarian goals: mental scanning practice; repetitive priming (non-stereotypical association, individuation and negation of stereotype)	Mental scanning can be applied as a long self-managed method. Priming was tested during exhaustive repetition mostly in labs	Behavioral self-regulation

## Section (C): Potential Uses of EVR for Training Empathy-Related Abilities

We next discuss different ways in which science and the arts have used VR in order to explore its potential in promoting empathy.

### Definitions and Description of VR

The term VR has been applied to different technologies with a variety of different characteristics that can be grouped in the following concepts:

(a) Non-immersive VR (Slater and Sanchez-Vives, 2016): refers to the use of tridimensional environments created by computer generated imagery that allows users to navigate in a virtual space. This technique uses two dimensional visual interfaces (such as computer screens and projectors).
(b) Immersive VR (Slater and Sanchez-Vives, 2016): refers to tridimensional environments with immersive visual interfaces such as VR glasses or immersive projections, such as the CAVE system (Cruz-Neira et al., 1993). Some of these technologies are more immersive than others. While VR headsets offer tridimensional images while eliminating any visual contact with the physical world, CAVE systems use a 360° field of mapped projections of a room, preserving the perspective of one’s own body. Videogenerated environments—usually monoscopic cameras with a 360° field of view—have also been increasingly used in immersive VR (Slater and Sanchez-Vives, 2016) especially in immersive journalism (Hardee and McMahan, 2017). Different from computer-generated environments, most of the content in this format limits users to a passive mode (Hardee and McMahan, 2017).

### Perceptual Illusions in VR

#### Presence [Place Illusion (PI) and Plausibility Illusion (Psi)] in VR: Being There and Feeling It Is Real

While the concept of “immersion” refers to the physical nature of a system, presence is its subjective correlative (Slater and Sanchez-Vives, 2016). The term presence has been used to convey many alternative meanings (Slater, 2009). In real life, “presence” is the state of being present (Hildebrandt et al., 2017) or is the state of existing in the world and, fundamentally, to have a body. In VR, the term presence is not necessarily related to having a body, but as the feeling of “being there” (Held and Durlach, 1992; Sheridan, 1992). This phenomenon has been referred to by scientists such as Mel Slater as “PI” in order to distinguish it from different concepts and is defined by “the strong illusion of being in a place in spite of the sure knowledge that you are not there.” Slater defines Psi as a different concept generally associated with presence. Psi stands for the illusion that the environment exhibited in VR is actually taking place. While PI is constrained by sensorimotor contingencies of the VR system, Psi relates to the credibility of the scenario. In both cases, users know that they are not “there” and that the events are not happening, but they feel as if they are, leading them to adopt behaviors as if they were really inhabiting the virtual environment (Slater and Sanchez-Vives, 2016). The interrelation of presence, engagement, and empathy has been observed in immersive VR experiences that teletransport the user to the environment of one emoter (Schutte and Stilinović, 2017).

#### Embodied VR or Full Body Ownership Illusion: Feeling That You Have a Different Body, With Different Traits

Immersive EVR or immersive VR with body ownership illusions (Maselli and Slater, 2013) refers to an adaptation of the technique of the Rubber Hand Illusion (Botvinick and Cohen, 1998) to create full body illusions in VR (Petkova and Ehrsson, 2008; Maselli and Slater, 2013). Using VR, researchers apply multisensory and motor stimuli in synchronicity with the first-person perspective of an avatar—using computer generated imaging (Maselli and Slater, 2013), or the image of real humans through stereoscopic video (Petkova and Ehrsson, 2008). In these studies, the evidence shows that subjects feel that they have swapped bodies with another person (Petkova and Ehrsson, 2008), a plastic mannequin (Petkova and Ehrsson, 2008), a Barbie doll (Van der Hoort et al., 2011), a digital avatar (Maselli and Slater, 2013), an invisible body (Guterstam et al., 2015), and even a body located in the front of them (Lenggenhager et al., 2007). These multisensory stimuli elicit a blurriness in the identity perception of self and other (Paladino et al., 2010) and may even drive participants to present a subjective anxiety to threats targeted at their virtual hand (Zhang and Hommel, 2016). Body Ownership, or the sense of embodiment, is comprised of the sense of self-location, the sense of agency and the sense of body ownership (Kilteni et al., 2012).

The most explored stimuli for inducing embodiment are visuomotor synchronicity, seeing oneself in the body of an avatar that mimics one’s movement in real time, and visuotactile synchronicity, seeing tactile stimuli applied to the avatar at the same time that it is applied to the hidden body part of the user (Maselli and Slater, 2013) with the avatar in a congruent posture with the subject (Tsakiris et al., 2007). Visuomotor synchronicity can be applied only to movements of the head, or also to movements of the whole body (Maselli and Slater, 2013), and visuotactile synchronicity can be passive (e.g., being touched) or active (e.g., touching an object) (Tajadura-Jiménez et al., 2013). Maselli and Slater (2013) have shown that a proper combination of stimuli to promote strong embodiment illusions that includes realistic images and wide field of view may not require visuomotor or visuotactile stimulation. In fact, incongruent perceptual cues may not break the embodiment during strong illusions. In one experiment, they showed that an avatar with realistic skin tone placed congruently to the user in immersive VR does not require visuomotor or visuotactile synchronicity to produce the embodiment. Moreover, full body visuomotor synchronicity using the image of an avatar with realistic skin tone can induce Body Ownership Illusion even under asynchronous visuotactile stimulation, which does not occur when the image of the avatar has a non-realistic skin tone (Maselli and Slater, 2013). Other combinations of stimuli, such as congruent full body first-person perspective and visuotactile synchronicity, can also be sufficient to create strong embodiment illusions, even with non-realistic human images (mannequins) and no head movements (Petkova and Ehrsson, 2008).

As well as visuomotor and visuotactile synchronicity, congruent first-person images, and realistic images, there are other variables that may induce or enhance manipulations in the perception of the body. Sound has been shown to alter the perception of the body, even without the use of VR. For example, altering the sound feedback when touching objects may alter the perception of the arm length (Tajadura-Jiménez et al., 2012, 2015a) and/or its strength (Tajadura-Jiménez et al., 2017). Similarly, manipulation of the sound feedback of a hammer hitting the user’s virtual hand can make the participants hand feel stiffer and heavier (Senna et al., 2014). Furthermore, manipulations of the sound of someone’s steps may also alter the perception of one’s own body weight and change the pattern of their gait (Tajadura-Jiménez et al., 2015b). Sound manipulation techniques have also been applied to EVR by changing the frequency of the user’s voice feedback to become more similar to the avatar (childlike) causing changes regarding the user’s voice recalibration toward the auditory feedback (Tajadura-Jiménez et al., 2017). These experiments show the potential of sound manipulation to enhance EVR experiences. Manipulations of interoceptive signals can also modulate embodied illusions. Evidence suggests that feedback of biosignals such as a heartbeat may enhance embodiment illusions (Suzuki et al., 2013). Synchronous cardiovisual signals increased self-identification and self-location in relation to the subject’s virtual body, shifting their perception of touch toward the virtual body (Aspell et al., 2013).

Recent theories of body cognition offer an interesting perspective on the potential nature of these processes. Tsakiris (2017) bases his model on extensive reviews and experiments, suggesting that one’s perception about one’s own body combines the interaction of bottom up brain phenomena (from the body to the brain) and top down processes (from the brain to the body). In this model, real time information of interoceptive states (such as proprioception, breathing, heart rate, and arousal) and real time information of exteroceptive sensations (such as vision, touch, and taste) inform the brain’s predictions of the perception of the body. The regulation between internal bodily states, external environment information, and mental concepts give us the sense of ourselves and the space that surrounds us. Neuroimaging research suggests that a significant prediction error is required to update the predictive internal models of the body matrix (O’Reilly et al., 2013; Riva et al., 2017).

Table 4 summarizes highlighted concepts related to Body Ownership Illusion.

**Table 4:** Highlighted variables and perceptive dimensions of Body Ownership Illusions.

Highlighted variables used in different combinations	Visuomotor synchronicity of head and/or body, visuotactile synchronicity (active or passive), congruent first person perspective, agency (partial or complete), realistic image, audio, and biosignals feedback

Perceptive dimensions	Attribution; self-location; agency

#### Agency Illusions

Although correlated, evidence suggests that Agency and Body Ownership are two different phenomena (Sato and Yasuda, 2005) that can occur in different circumstances. Agency requires voluntary action, while body ownership may occur under both voluntary action and passive events (Tsakiris et al., 2007). The subjective perception of agency over a body part is different from the subjective perception of agency over a physical action, that by being voluntary, involves a combination of efferent (top down) and afferent (bottom up) information (Tsakiris et al., 2007). The rubber hand illusion with visuotactile stimulation is a classic example of Body Ownership Illusion without agency (Botvinick and Cohen, 1998), that actually involves a subjective perception of agency, with affirmative agreements with the sentences “it seemed like I could have moved the rubber hand if I had wanted” and “it seemed like I was in control of the rubber hand” in self-reported questionnaires used to measure the illusion. But this subjective perception of agency decreased, for example, with visuomotor delay, without changing the sense of ownership (Kalckert and Ehrsson, 2012). Even though it is not necessary for inducing Body Ownership Illusions, agency may contribute to the extent that this illusion is felt. Using proprioceptive drift, the perception that the real hand is closer to the displaced rubber hand, Tsakiris et al. (2006) suggest that voluntary movements of body parts induce a global change in proprioceptive awareness. While localized proprioceptive drifts were found in passive stimulation, during active movement of one digit the proprioceptive drift was observed in the whole hand.

More recently, different studies have used the Body Ownership Illusion with visuomotor synchronicity and voluntary movement to induce an illusion of agency over the user actions such as walking (Kokkinara et al., 2016) or speaking (Banakou and Slater, 2014). By embodying a digital avatar that could be controlled by the user’s movements, researchers observed self-attribution of agency to subjects over actions taken by the avatar, even without any prior intention, prediction, priming, and cause preceding effect (Banakou and Slater, 2014). In this experiment, the digital avatar would speak independently of the user’s action creating not only the perception that subjects were themselves talking, but also changing the fundamental frequency of the user’s voice after the experience. This illusion was found to be even stronger when a vibration stimulus was applied to the user’s throat in synchronicity with the avatar’s voice.

As with the model of body cognition discussed in Section “Embodied VR or Full Body Ownership Illusion: Feeling That You Have a Different Body, with Different Traits,” several theories define the sense of agency as a result of the comparison between prediction of efferent and afferent information. For a cognitive and neural perspective of the sense of agency see the review of David et al. (2008). These concepts were implemented in an experiment in which researchers were able to induce the illusion of walking in subjects who were actually seated (Kokkinara et al., 2016) through a combination of priming and body ownership illusion. In this experiment, subjects could see themselves walking while perceiving an optic flow in the environment and a sway movement of the head due to the walking motion. Subjects presented high levels of body ownership in self-report questionnaires and based on physiological data.

Taken together, these findings suggest that Agency Illusions could be combined with Body Ownership Illusions to create experiences in which the avatars perform prosocial actions that could be perceived as voluntary actions of the subjects themselves. Even so, this hypothesis is yet to be investigated before being implemented into empathy-related training.

### Using Perceptual Illusions to Promote Empathy-Related Abilities

Since the 2000s VR has been used to study perspective taking (Gaunet et al., 2001; Lambrey et al., 2012). VR allows users to move their perspectives to different scenarios and universes. One can furthermore play different roles from the perspective of different avatars. The ability of immersive VR to displace the first-person point of view relates directly to perspective taking and role playing. Experiences intended to promote empathy-related abilities have been developed in both types of VR. Some iconic examples are the non-immersive VR Game Real Lives (Bachen et al., 2012), the immersive VR 360° video projects from the United Nations, *Clouds Over Sidra* (Schutte and Stilinović, 2017), and The New York Times project, *The Displaced* (Sirkkunen et al., 2016). In these last two examples, presence has been correlated to positive empathic responses, showing the power of this media to engage users and attract their full attention on the stories of other individuals. Although clearly powerful, these examples place the viewer in the third person perspective. As the previous pages suggest, VR can amplify these effects by using its full potential to place users in the first-person perspective of the other. Being in the first-person perspective of an avatar that moves in synchronicity with the user may help participants to overcome cognitive loads increasing their memory performance after the VR experience (Steed et al., 2017). Tentative evidence of the positive effect in embodiment and presence of having the first-person perspective of an avatar have also been observed in experiments in uncontrolled settings conducted remotely through an APP through the Internet (Steed et al., 2016). Having an avatar also places participants into the center of the experience (de la Peña et al., 2010). For more examples of immersive VR approaches without the use of embodiment, see the review of Hardee and McMahan (2017) on immersive journalism.

Experiences of EVR allow users to literally step into the shoes of others and see the world from their perspective. Research on EVR has explored how manipulations of the senses can be used to modulate empathic responses. Experiences of stepping into the shoes of outgroup members have shown significant plasticity of empathic abilities even after the experience by decreasing implicit racial biases (Peck et al., 2013) and increasing of mimicry of outgroup members (Hasler et al., 2017). EVR may also affect an individual’s self-concept and behavior *via* the traits (positive or negative) of the characters represented in their avatars (Yee and Bailenson, 2007). For example, subjects who embodied a super-hero increased their altruistic intentions more than subjects that have embodied a super villain (Rosenberg et al., 2013).

#### Multisensory Perspective Taking of Outgroup Members: Affecting Bias, Mimicry, Perception of Similarity, and Emotion

Some experiments have used EVR to allow participants to step into the shoes of outgroup members. Peck et al. (2013) conducted a study in which subjects with light skin could see themselves in a dark-skinned avatar. The manipulation decreased negative implicit associations toward black individuals immediately after the experiment. A similar setting was conducted by Banakou et al. (2016) showing that a decrease of implicit bias was sustained even 1 week after the intervention. In another experiment (Hasler et al., 2017), levels of implicit biases did not change, but the intervention nevertheless increased mimicry between a subject with white skin embodying an avatar with dark skin and another digital character with dark skin.

In one non-experimental setting, de la Peña et al. (2010) used the concepts of multisensory perspective to allow participants to step into the shoes of a character confined in a Guantanamo Bay. Visuomotor synchronicity of the user’s head, binaural audio of the environment and haptic feedback of breathing were provided to create the illusion of being in the stress position of the prisoners (a position described in reports on prisoner treatment). Although no scientific data were collected, participants reported feeling anxiety and discomfort and expressed an emotional connection with the situation of the prisoners.

Using a different approach and without immersive interfaces, researchers used a different technique called “enfacement,” that stimulates a mirror touch synesthesia (Fini et al., 2013). Participants see the image of faces of different avatars on screen being stroked by a brush. Mimicking the tactile stimuli provided in the avatar, researchers stroked the user’s face with an identical brush, in synchrony with the image observed. After seeing the image of avatars with different phenotypes, subjects revealed a greater self-identification with more diverse phenotypic characteristics. Using enfacement illusions with EEG measurements, Serino et al. (2015) observed activation of face-specific regions correlating to the increased identification with the avatar’s face.

#### Manipulation of Interoceptive Signals: Affecting Emotion Regulation

In a recent experiment without VR (Azevedo et al., 2017a,b), researchers showed that tactile feedback of a slow heartbeat-like rhythm can make subjects more relaxed before performing a stressful task such as speaking in public, showing its potential implications in emotion regulation (an important ability for converting empathic distress into empathic concern). As mentioned in Section “Embodied VR or Full Body Ownership Illusion: Feeling That You Have a Different Body, with Different Traits,” interoceptive signals can interfere in embodiment. It has also been shown that interoceptive signals such as heartbeat can correspond to bias behavior (Azevedo et al., 2017a,b) and that awareness of heartbeat signals relate in significant part to cognitive-affective processing (Dunn et al., 2010). These findings reveal a great potential for integrating biosignal manipulations to help participants to control their anxiety when faced with the stress of others, to interfere in anxiety triggered by an outgroup threat and to interfere in emotional processing such as affective empathy.

#### Proteus Effect: Affecting Stereotypes, Self-Perception, and Behavior

In virtual environments, digital self-representations (i.e., avatars) may influence users and lead their behaviors to be consistent with the avatar’s appearance. This behavioral modulation, known as the Proteus Effect (Yee and Bailenson, 2007; Yee et al., 2009), has been observed in several studies. In their seminal work, Yee and Bailenson (2007) have shown that attractive avatars lead to a more intimate behavior with a confederate in terms of self-disclosure and interpersonal distance. In a second study, they also observed that tall avatars lead to more confident behavior than short avatars in a negotiation task. More recent studies have also shown that the appearance of the embodied avatars could influence attitudes, beliefs (Fox et al., 2013) and actions of the users (Peña et al., 2009; Guegan et al., 2016). For instance, it has been shown in non-immersive VR environments that the use of an avatar resembling a member of the Ku Klux Klan activates more negative thoughts and leads users to participate more aggressively (e.g., murder, vengeance) in the stories created (Peña et al., 2009). Other studies have shown how the Proteus Effect may enhance stereotypes against outgroup members. It has also been shown that users that play a black avatar in a computer game present greater aggressive cognition and affect (Eastin et al., 2009; Ash, 2016).

From a theoretical point of view, the Proteus effect is based on self-perception principles (Bem, 1972), under which the individual explains his attitudes and internal states based on observation of external cues. In this way, the profile of the avatar could lead the user to make implicit inferences about his/her personal dispositions (e.g., I am an empathic person). The influence of avatars is also compatible with the priming process (Peña et al., 2009), which refers to “the incidental activation of knowledge structures, such as trait concepts and stereotypes, by the current situational context” (Bargh et al., 1996). For instance, perceiving the characteristics of an avatar nurse or a humanitarian worker could activate some related concepts (e.g., altruism, empathy) as well as inhibit more antithetical concepts such as aggression or violence. Moreover, these situational cues may lead to behavioral assimilation *via* an increase in the likelihood of behaviors congruent with the primed concept. Whatever the underlying mechanism, and to the best of our knowledge, no study has directly investigated the links between the Proteus Effect and the empathic processes. However, previous work shows that the digital self-representations can influence behavior in a pro or antisocial way. For example, the embodiment of an avatar resembling an inventor led engineering students to show higher creative fluency and originality of ideas during a face-to-face brainstorming session conducted after immersion in a virtual environment (Guegan et al., 2016). Another example shows that embodying a casually dressed black avatar enhances users’ performance in playing drums in comparison to embodying a formally dressed white avatar (Kilteni et al., 2013), probably due to the positive stereotypic association of black individuals and rhythm. In another experiment, it was demonstrated that embodying a Sigmund Freud-like avatar talking to a scanned version of their own body can help users improve their mood after self-counseling, in comparison to self-counseling in a self-representing avatar (Osimo et al., 2015). Another case of positive stereotyping showed that embodying a superhero who helps the population of a virtual city led to increased prosocial behavior in an offline interaction (Rosenberg et al., 2013). Conversely, using a villainous avatar (Voldemort) led both to an increase in antisocial behavior and to a significant decrease in prosocial behavior (Yoon and Vargas, 2014).

It has been shown that the Proteus effect is mediated by the level of embodiment felt by users in relation to their avatar (Ash, 2016), suggesting that EVR can enhance this effect. Given this set of findings, one might expect that training methods using avatars designed and pretested to improve empathy would induce beneficial behavioral changes, improve positive perceptions among users, and so on. Another possible implication of this concept is to embody a digital avatar of an outgroup member that presents traits that contradict stereotypes. These hypotheses have yet to be tested.

Table 5 summarizes highlighted strategies that can be applied using VR and Body Ownership Illusion for empathy learning and their potential effects.

**Table 5:** Highlighted concepts of embodied virtual reality that can be used for empathy-related training.

EVR strategies	Place illusion and plausibility illusion	Body ownership illusion	Agency illusion	Interoceptive signals manipulation	Proteus effect
Techniques	Sensorimotor stimuli and highly credible environment	Multisensory and motor perspective taking	Embodiment combining voluntary and involuntary actions	Manipulated feedback of interoceptive signals (decreased heart beat)	Avatars presenting empathy-related traits and appearances

Expected responses in users	Behaving and feeling as if they were in the VR environment	Modulation of bias, mimicry, similarity and emotion after EVR experience	Self-attribution of avatar’s actions	Distress regulation	Reinforcement of stereotypes (positive or negative); modulation of self-perception and behavior after the EVR experience

### The Machine To Be Another: An Artistic Exploration of EVR Methods for Learning Empathy

To extend the examples beyond scientific fields, the article will briefly describe one artistic system in VR, designed to promote empathy-related behavior, called The Machine to Be Another (TMTBA; Bertrand et al., 2014; Sutherland, 2014; Oliveira et al., 2016), created by one of the authors of this article together with the interdisciplinary collective BeAnotherLab. Although there are no scientific results of these experiments in promoting empathy, the system adopts interesting approaches to address its goals. Inspired by embodiment studies, TMTBA allows users to see themselves in the body of real human beings (captured by video) instead of using computer generated images. The group uses different technological sets, the most famous being “Body Swap.” In this installation, two users swap perspectives using VR headsets and first-person cameras, while being instructed to move slowly and to collaborate with each other in order to synchronize their movements while the artists provide physical interactions to stimulate touch. Besides swapping perspectives (under visuomotor and visuotactile synchronicity) the Body Swap is used to present real narratives from different individuals acting as performers. Over a 5-year period, the group has presented several performances from individuals such as asylum seekers in a detention center in Israel, an Iraq veteran in the USA, an African migrant in Spain and victims of police brutality in Brazil. These narratives are created by performers themselves, drawing on their personal views on subjects—from social stigma to stories of forgiveness. The use of real people with real stories, is possibly the main conceptual difference of this artistic work to other lab studies. After 10 min of physical interaction—exploring movements of their hands, arms and legs, as well as interacting with physical objects and with mirrors—they are placed face to face, similar to Petkova and Ehrsson’s experiment (Petkova and Ehrsson, 2008) in which one person can shake hands with their own selves, from the perspective of another individual. Another interesting aspect of the system is that it allows the performer and user to meet physically, immediately after the VR experiment, something that would not have taken place in the everyday life. TMTBA has been broadly presented in over 25 countries in artistic, cultural, and academic contexts, and is used as a tool to promote mutual understanding. These presentations have taken experiments based on concepts of Body Ownership illusion to a wide range of audiences, enabling them to experience the perspective of another real human being in the routine of their lives. Designed with a low-budget, this system has several limitations in comparison to other lab studies of embodiment, such as: the use of a monoscopic camera, constrained field of view, and lower resolution due to the quality of the hardware used (initially Oculus Rift Dk2). Even so, the group has collected anecdotal evidence from user’s statements, many of which have been reported in the press (EL PAIS*; TV GloboNews**; The Verge***) (Souppouris, 2014; María, 2016; Cristina, 2017). In users’ statements, subjects report concern toward performers, pointing to the potential use of this type of experience as an immersive media for social interaction. In order to clarify the effectiveness of these systems and protocols, controlled studies have yet to be developed.

**Figure 1:**
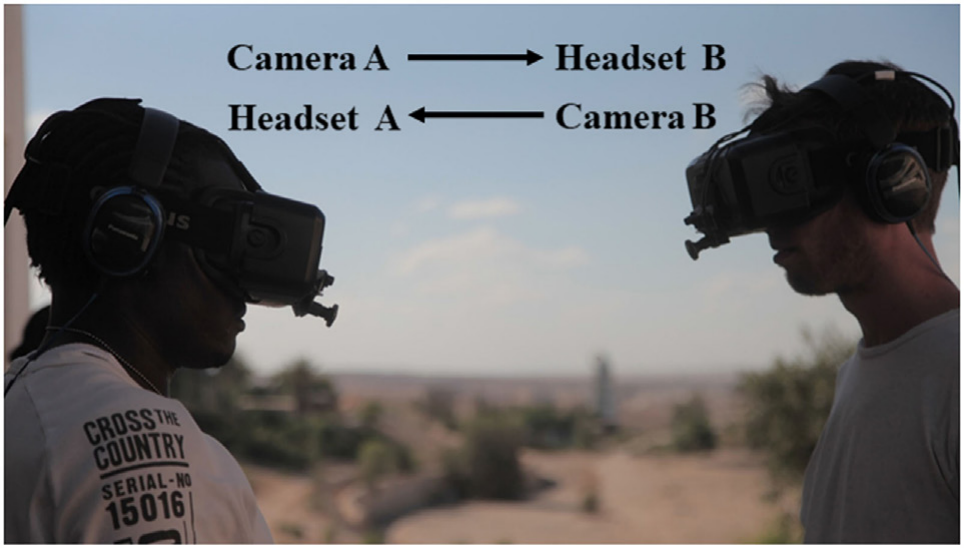
Functional diagram of two users swapping bodies through the system The Machine to Be Another. In this interactive mode, both users have to mirror each other in order to move in syncrony. Picture from workshop held by BeAnotherLab in 2015 at detention center for asylum seekers in Israel.

**Figure 2:**
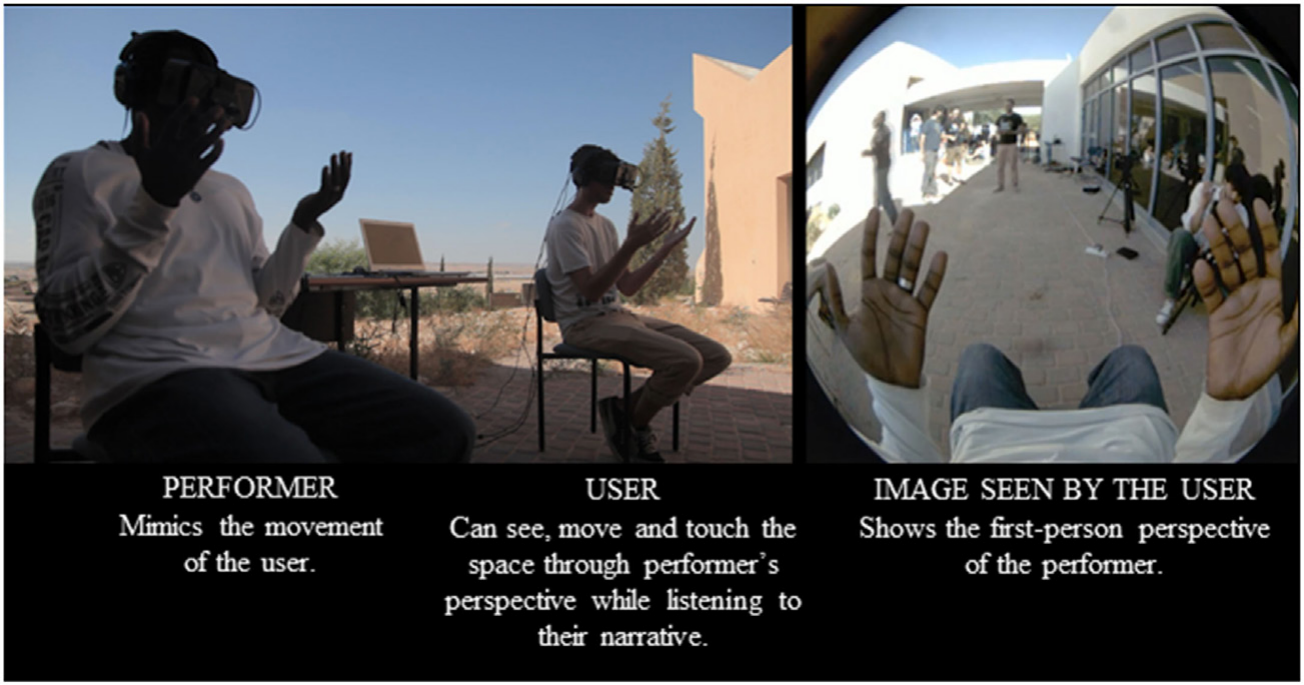
Functional diagram of performance using the The Machine to Be Another. Picture from workshop held by BeAnotherLab in 2015 at detention center for asylum seekers in Holot (Israel), presenting the narrative of Drhassn steib.

Figures 1 and 2 document a workshop held by Beanotherlab in a detention center for asylum seekers in Israel with functional diagrams of the different interactive modes of the body swap experience.

## Discussion

This review article has brought an interdisciplinary perspective to promote insights on how to use VR for training empathic skills. It offers a guide for highlighted concepts, educational practices and VR techniques that can be used in empathy-related learning. The referred literature is recommended for deeper understanding each of these complex topics.

The collaborative aspects of constructivist approach could also offer possibilities of interaction with outgroup characters in VR. The constructivist focus on building self-reflexivity could also be incorporated into the idea of mapping and controlling coded predictions. By inviting learners to understand their own misconceptions, strategies of non-stereotypic information could be used as part of the process of the intervention. This training method could therefore be focused on provoking intergroup encounters (in VR and real life) in a series of interventions using EVR, in which subjects can experience in EVR the perspective of an outgroup (multisensory role playing of an outgroup). Explorations of Proteus Effect could be applied through non-stereotypical information revealing more of the context and experiences of the other (individuation). Subjects could then be exposed to real life situations (situated learning) of prejudices faced by outgroup members. This could offer subjects a better idea of the challenges faced by stigmatized outgroups (familiarity). Moreover, in theory we could induce empathy through an empathic personality of the avatar that could demonstrate compassionate discourse (compassionate avatar) and engage in altruistic behavior (altruistic avatar). In order to enhance the experience, manipulations of biofeedback information such as heartbeat and breathing (interoceptive manipulation) could be used to help subjects control anxiety (emotion regulation of distress). By promoting the perception of presence (place and Psi), the experience would likely raise the subject’s awareness to the events of the simulation, possibly enhancing their capabilities of self-reflection. A similar methodology could be used to place subjects in different situations under the perspective of one ingroup member, or an avatar similar to themselves. These situations could explore, for example, a task in which they must collaborate with an outgroup member (intergroup collaboration and familiarity), or where they would be helped by an outgroup member (non-stereotypic information). It could also explore the perspective of one observer facing an intergroup interaction between one dominant and one stigmatized individual, in which the stigmatized individual has something in common with them (e.g., a T-shirt of their football team), or having the possibility to help or being induced to help (altruistic agency illusion).

These are some ideas of experiments that would apply existing knowledge to concrete training methods. Each of these examples raises several research questions:

– Can EVR facilitate collaborative intergroup encounters?
– Can the Proteus effect be used to trigger non-stereotypical information about outgroup members, as well as compassion, altruism and egalitarian goals?
– Can non-stereotypical traits of an avatar in EVR help learners to overcome stereotypes?
– Can agency illusions be used to induce self-attribution of prosocial actions targeted to outgroup members?
– Can long-term training in EVR produce long-term changes in empathy?
– Can emotion regulation be enhanced by EVR experiences with interoceptive manipulations?
– Can mindfulness practice be enhanced by presence (PI) in VR?

These are just a very few questions that demonstrate a fertile universe for research and for integration of EVR with training methods for empathy-related abilities. With the current democratization of VR devices, the use of EVR has become more accessible, making it possible to develop EVR training methods that can be implemented outside the lab, in contexts such as educational, cultural and artistic environments. Research on these techniques could open doors for the design of new learning tools that, if effective, could have a wide effect in promoting a more empathic society.

## Recommended Strategies for New Learning Applications of Empathy-Related Abilities in EVR: Proposed Equalizing Model and Framework for Empathy Learning

In this section, we will discuss how to integrate the content summarized in this article in the design of EVR based empathy training programs.

As it was demonstrated, empathy-related responses are result of a complex phenomenon that involves different intergroup, interpersonal and intrapersonal processes and mechanisms. This means that there is no single recipe for empathy development and that several variables in the social environment of the interaction may interfere in what is the most appropriate ability that needs to be developed. Therefore, the first step we recommend is one analyses of all factors related to the social environment of the interaction aiming to stimulate optimal empathic processes: positive intergroup interaction and evaluation, awareness of the other, awareness of the self, empathic concern and altruist behavior. These processes can be analyzed guided by the following questions: (1) What is the relationship between emoter and observer? (2) How developed is the self-awareness of the observer? (3) How developed are the empathic abilities of the observer toward the emoter?

To help to identify what type of ability needs to be enhanced, we also propose a framework that relates each of these questions to a list of relevant abilities, catalysts, and moderators as well as effective learning methods and EVR strategies that can be used. Even so, abilities, catalysts, moderators, methods, and EVR strategies may interconnect beyond this division, spilling over effect into other dimensions.

Table 6 presents a framework with the most relevant concepts discussed in this article, guiding the design of learning systems within all domains of the equalizer (social interaction context, variables, and enhancer).

**Table 6:** Framework for equalize empathic processes and expressions through learning methods and embodied virtual reality, based on highlighted concepts and practices.

(1) **What is the relationship between emoter and observer?** *Abilities*: intergroup openness; reflexive thinking; social skills; conflict management. *Catalysts*: Long-term training, safe environment collaborative dynamics; engaging voluntary activities	

*Modulators*: increase of familiarity, affiliation, similarity with outgroup members; decrease of bias, stereotypes, coded predictions, categorical thinking against outgroup members; enhancement of egalitarian goals and self-analysis of-group fairness related to outgroup members	

*Learning methods*: Constructivism and SEL for instrumentalization of reflexive thinking and social skills; implementation of egalitarian goals: repetitive priming for non-stereotypical association, individuation and negation of stereotype; mindfulness training for practice of non-judgmental thinking	*EVR methods*: intergroup embodiment for enhancing self-other similarity; proteus effect with non-stereotypical avatar

**(2) How developed is the self-awareness of the observer?** *Abilities*: bodily, emotional, cognitive, and social self-awareness. *Catalysts*: Educators as facilitators	

*Modulation*: self-other distinction; emotion recognition; egalitarian internal, and social goals	

*Learning methods*: mindfulness training for interoceptive awareness; implementation of egalitarian goals: mental scanning	*EVR methods*: PI and PSI Illusion; interoceptive feedback of heart beats

(3) **How developed are the empathic abilities of the observer toward the emoter?**	
*Abilities*: affective empathy; cognitive empathy; empathy accuracy; empathic distress moderation; compassion; altruism; problem solving. *Catalysts*: Real world case based and contextual knowledge	

*Moderators*: emotional engagement; perspective taking; online simulation; dialog skills; present attention; loving-kindness; motivation, power and skills for helping; self-regulation of behavioral expressions	

*Learning methods*: role-playing; mindfulness training for present attention, perspective taking and compassion and for decreasing anxiety; implementation of egalitarian goals: mental scanning	*EVR methods*: multisensorimotor first-person perspective taking synchronicity; proteus effect and/or agency illusions of compassionate and prosocial avatars

*Obs.: highlighted abilities, moderators, and methods may interconnect and overlap*.

After a contextual analysis based on this framework, it will be possible to define the most relevant empathic abilities, catalysts factors, and moderators that can act as important variables of the empathic process in one given situation, and therefore, to define the structure of the learning system. Once the variables that need to be addressed are clear, it is possible to choose relevant enhancers of these variables: training methods and EVR strategies. These enhancers will interfere in one another, what also must be calibrated focusing the structure of the learning system defined in the previous steps. Following these steps will allow the design of learning systems that make an efficient use of relevant EVR strategies.

Training methods can combine several abilities, modulators, catalysts, practices and EVR strategies aimed at promoting an optimal empathic response in subjects. Through this framework, we expect to offer educators insights into different strategies that could be adopted to help learners to develop skills for building a world of tolerance and mutual understanding.

## Author Contributions

PB developed conception of the structure, performed search for references, analyzed and edited data, wrote the first draft, and contributed to the development of the manuscript till last editions; JG wrote the section on Proteus Effect; LR contributed to references and revision of sections regarding Empathy and Training Methods; CM contributed to the section on Empathy offering references and critical revision under the perspective of social neuroscience, as well as contributing to the critical revision of the whole article; FZ contributed to the whole process writing sections, providing references, and performing critical revision and final edition.

## Conflict of Interest Statement

The first author is also cocreator of one art/research work mentioned in the article—The Machine to Be Another—and cofounder of the non-for-profit cultural association BeAnotherLab that presents this work in several contexts. All other authors declare that the research was conducted in the absence of any commercial or financial relationships that could be construed as a potential conflict of interest.
